# From relaxed beliefs under psychedelics (REBUS) to revised beliefs after psychedelics (REBAS)

**DOI:** 10.1038/s41598-023-28111-3

**Published:** 2025-01-29

**Authors:** Richard J. Zeifman, Meg J. Spriggs, Hannes Kettner, Taylor Lyons, Fernando E. Rosas, Pedro A. M. Mediano, David Erritzoe, Robin L. Carhart-Harris

**Affiliations:** 1 Centre for Psychedelic Research, Department of Brain Sciences, Faculty of Medicine, Imperial College London, London, UK; 2 NYU Langone Center for Psychedelic Medicine, NYU Grossman School of Medicine, 1 Park Avenue, Floor 8, New York, NY 10016 USA; 3 Department of Informatics, University of Sussex, Brighton, UK; 4 Centre for Complexity Science, Imperial College London, London, UK; 5 Center for Eudaimonia and Human Flourishing, University of Oxford, Oxford, UK; 6 Department of Computing, Imperial College London, London, UK; 7 Department of Psychology, University of Cambridge, Cambridge, UK; 8 Psychedelics Division, Neuroscape, University of California, San Francisco, USA

**Keywords:** Human behaviour, Clinical pharmacology

## Abstract

The Relaxed Beliefs Under pSychedelics (REBUS) model proposes that serotonergic psychedelics decrease the precision weighting of neurobiologically-encoded beliefs. We conducted a preliminary examination of two psychological assumptions of REBUS: (a) psychedelics foster acute relaxation and post-acute revision of confidence in mental-health-relevant beliefs; which (b) facilitate positive therapeutic outcomes and are associated with the entropy of EEG signals. Healthy individuals (*N* = 11) were administered 1 mg and 25 mg psilocybin 4-weeks apart. Confidence ratings for personally held beliefs were obtained before, during, and 4-weeks post-psilocybin. Acute entropy and subjective experiences were measured, as was well-being (before and 4-weeks post-psilocybin). Confidence in negative self-beliefs decreased following 25 mg psilocybin. Entropy and subjective effects under 25 mg psilocybin correlated with decreases in negative self-belief confidence (acutely and at 4-weeks). Particularly strong evidence was seen for a relationship between decreases in negative self-belief confidence and increases in well-being. We report the first empirical evidence that the relaxation and revision of negative self-belief confidence mediates psilocybin's positive psychological outcomes, and provide tentative evidence for a neuronal mechanism, namely, increased neuronal entropy. Replication within larger and clinical samples is necessary. We also introduce a new measure for examining the robustness of these preliminary findings and the utility of the REBUS model.

## Introduction

Psychedelic therapy is receiving increasing attention for its putative transdiagnostic action across mental health outcomes^1^, including decreasing anxiety, depression, and suicidal ideation within clinical populations (e.g.,^2–12^), and enhancing well-being among healthy individuals^13–15^. In line with precision psychiatry^16^ and process-based psychotherapy^17^, identifying the mechanisms through which psychedelic therapy leads to positive therapeutic change may help to tailor, deliver, and maximise its therapeutic efficacy (see^18^).

The RElaxed Beliefs Under pSychedelics (REBUS) model^19,20^ provides a unified theoretical account of the effects of psychedelics and the mechanisms through which they lead to positive therapeutic outcomes. Building on the Bayesian hierarchical predictive processing view of brain function^21^, REBUS proposes that the “entropic” brain state induced by psychedelics^22,23^ corresponds to a relaxation in the precision-weighting of neuronally encoded predictive models. REBUS also proposes that within the transient drug-induced state of enhanced neural and cognitive plasticity, there may be a reduction in the felt confidence associated with maladaptive beliefs and assumptions (that are ordinarily encoded with excessive precision-weighting and confidence; e.g., negative self-perceptions), and increased amenability to therapeutic change.

Neurobiological support for REBUS can be seen in multiple experimental findings, including reduced hierarchical organisation and top-down processing in the brain (e.g.,^24–26^). Perhaps most notably, neuroimaging experiments with psychedelics have revealed a consistent increase in markers of neural entropy^27,28^, which predict behavioural reports during the experience^29^. This growing body of research contrasts with the lack of psychological research on the topic, which has not yet examined the effects of psychedelics on acute belief confidence, and whether this belief relaxation can serve as an opportunity for subsequent revision of these negative (self)beliefs and improved well-being; an important assumption of REBUS.

A key question related to the therapeutic application of psychedelics is how this putative acute relaxation translates into long-term therapeutic change. We refer to the post-acute revision of overweighted beliefs as REvised Beliefs After pSychedelics (REBAS) and propose that this is a fundamental feature of recovery from a broad swathe of mental illness. Within clinical and healthy populations, the administration of psychedelics is associated with decreases in negative attitudes toward the future^6,30–32^, which correlate with reductions in depression severity^32^ and suicidal ideation^7^. Similarly, the administration of a psychedelic is associated with self-reported increases in positive self-related beliefs (e.g.,^31,33,34^). However, research has not yet examined whether administration of a psychedelic leads to decreases in negative self-beliefs. Furthermore, research has been limited by the use of nomothetic (i.e., general) rather than idiographic (i.e., personally identified) measurement of self-related beliefs (for the benefits of idiographic approaches, see^35^). Accordingly, evidence supporting a relationship between negative belief relaxation and revision and neurobiological markers of REBUS (e.g., increased neural entropy as a corollary of relaxed precision weighting) or subsequent improvements in well-being remain elusive.

REBUS offers an account of much of the phenomenology of the psychedelic experience, including the unitive experience where discriminative beliefs diminish and are replaced by a sense of reciprocal interconnectedness^36^. Research supports a role for subjective unitive experiences in mediating long-term outcomes after psychedelics^37^, as well as an association between neurobiological indices of REBUS (such as decreased top-down information flow and hierarchical organization in the brain) and properties of the acute experience—including the unitive experience^24,38,39^. However, research has not yet directly examined psychological indices of belief relaxation under psychedelics (REBUS), and revision after psychedelics (REBAS), nor whether these relate to the intensity of the acute unitive experiences elicited under psychedelics or subsequent enduring psychological changes.

In sum, the REBUS model suggests that psychedelics can potentially improve mental health by *relaxing* and *revising* the confidence associated with overweighted beliefs. However, there is still no evidence as to whether: (a) psychedelics acutely decrease confidence in (i.e. *relax*) negative self-beliefs; and (b) induce lasting decreases in negative self-belief confidence (i.e. *revision*); and (c) this *relaxation* and *revision* of overweighted beliefs are associated with neurobiological markers of REBUS, acute unitive experience, and long-term improvements in mental health. Therefore, in this paper we examined whether:

1. Administration of psilocybin leads to decreases in felt confidence in negative self-beliefs (a) acutely (*belief relaxation; REBUS*) and (b) 4-weeks later (*belief revision; REBAS*).
2. *Relaxation* (REBUS) and *revision* (REBAS) of confidence associated with negative self-beliefs are associated with: (a) neurobiological markers of REBUS (i.e., entropy), (b) the intensity of the acute unitive experience, and (c) increases in well-being 4-weeks after psilocybin administration.

## Results

### Self and other (negative/positive) belief confidence

**Figure 1:**
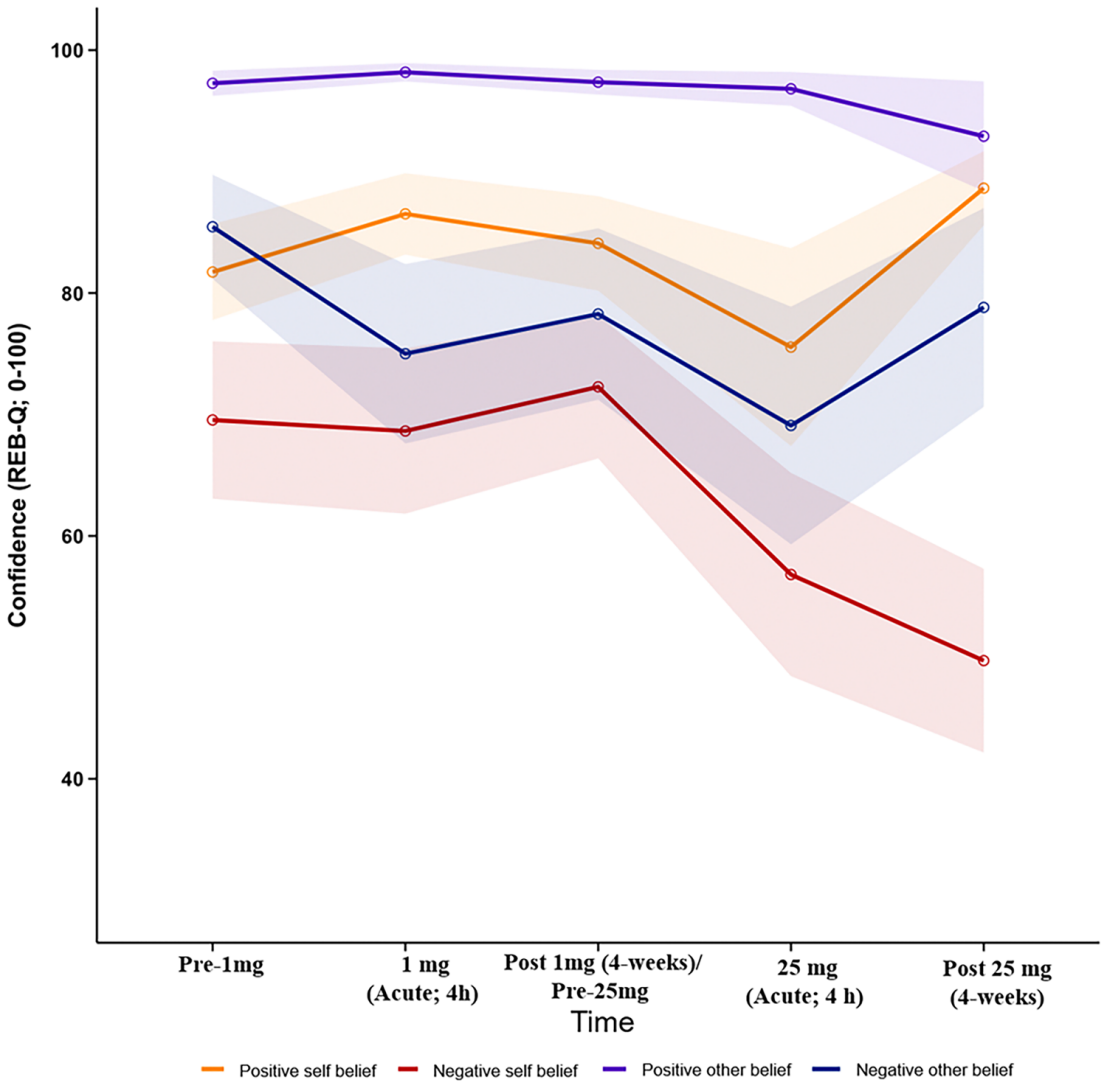
Changes in belief confidence following 1 and 25 mg psilocybin administration. Shaded areas represent standard errors. Acute confidence ratings were collected 4 h after administration of psilocybin.

For results of ANOVAs, effect sizes, and Bayes Factors examining changes in confidence related to participants’ beliefs, see Table 1. For changes in belief confidence over time, see Fig. 1. A significant effect of time (anecdotal evidence) was observed for negative other-belief confidence following 1 mg psilocybin with significant (anecdotal evidence) acute reductions in confidence. A significant effect of time (moderate evidence) was observed for negative self-belief confidence following 25 mg psilocybin with significant (moderate evidence) reductions in confidence 4-weeks after 25 mg psilocybin. No other significant effects of time were observed across belief categories for 1 mg and 25 mg psilocybin. However, a significant (substantial evidence) increase in positive self-belief confidence was observed 4-weeks after 25 mg psilocybin.

**Table 1:** Changes in belief confidence over time, means, standard deviations, effect sizes, and Bayes factors.

Belief type		1 mg psilocybin			25 mg psilocybin		
Negative self-belief		F(2, 20) = 0.273, *p* = 0.678, BF_10_ = 0.237^^^			F(2, 20) = 5.42, *p* = 0.013*, BF_10_ = 4.604^^^		
		Baseline	Acute	Post-acute	Baseline	Acute	Post-acute
	Mean	69.55	68.64	72.27	72.27	56.82	**49.73
	*SD*	21.50	22.59	19.54	19.54	27.77	25.11
	Cohen’s *d*_*z*_	–	0.05	0.17	–	0.54	0.98
	95% CI	–	− 12.52 to 14.34	− 8.14 to 13.60	–	− 3.81 to 34.72	7.14 to 37.95
	BF_10_	–	0.300^^^	0.3400		0.990	6.861^^^
Positive self-belief		F(2, 20) = 0.913, *p* = 0.417, BF_10_ = 0.356			^#^F(1.16, 11.59) = 3.69, *p* = 0.076, BF_10_ = 1.778		
		Baseline	Acute	Post-acute	Baseline	Acute	Post-acute
	Mean	81.73	86.52	84.09	84.09	75.55	*88.64
	*SD*	13.11	11.11	12.91	12.91	27.02	10.08
	Cohen’s *d*_*z*_	–	0.32	0.25	–	0.37	0.83
	95% CI	–	− 5.18 to 14.76	− 4.03 to 8.75	–	− 7.11 to 24.20	0.87 to 8.22
	BF_10_	–	0.477	0.396		0.667	3.453^^^
Negative other-belief		F(2, 20) = 3.93, *p* = 0.036*, BF_10_ = 1.916			F(2, 20) = 1.80, *p* = 0.192, BF_10_ = 0.597		
		Baseline	Acute	Post-acute	Baseline	Acute	Post-acute
	Mean	85.46	*75.00	78.27	78.27	69.09	78.82
	*SD*	14.22	24.50	23.41	23.41	32.47	27.17
	Cohen’s *d*_*z*_	–	0.74	0.56	–	0.63	0.03
	95% CI	–	1.01 to 19.90	− 1.45 to 15.81	–	− 0.65 to 19.02	− 10.41 to 11.50
	BF_10_	–	2.340	1.074		1.410	0.299^^^
Positive other-belief		F(2, 20) = 0.89, *p* = 0.425, BF_10_ = 0.350			^+^F(1.02, 10.24) = 1.04, *p* = 0.373, BF_10_ = 0.417		
		Baseline	Acute	Post-acute	Baseline	Acute	Post-acute
	Mean	97.27	98.18	97.36	97.36	96.82	92.91
	*SD*	3.44	2.52	3.39	3.39	4.62	14.99
	Cohen’s *d*_*z*_	–	0.30	0.04	–	0.36	0.33
	95% CI	–	− 2.93 to 1.12	− 1.43 to 1.61	–	− 0.47 to 1.56	− 4.70 to 13.61
	BF_10_	–	0.450	0.300^^^		0.533	0.482

*p < 0.05; **p < 0.01; ^^^strong evidence in support of the alternative (BF > 3) or the null (B < 0.3); ^#^Huynh–Feldt correction applied due to violation of assumption of sphericity based on Mauchly's Sphericity Test (ε = 0.580, *p* < 0.001); ^+^Huynh–Feldt correction applied due to violation of assumption of sphericity based on Mauchly's Sphericity Test (ε = 0.512, *p* < 0.001).

### Changes in negative-self belief confidence and oceanic boundlessness

**Figure 2:**
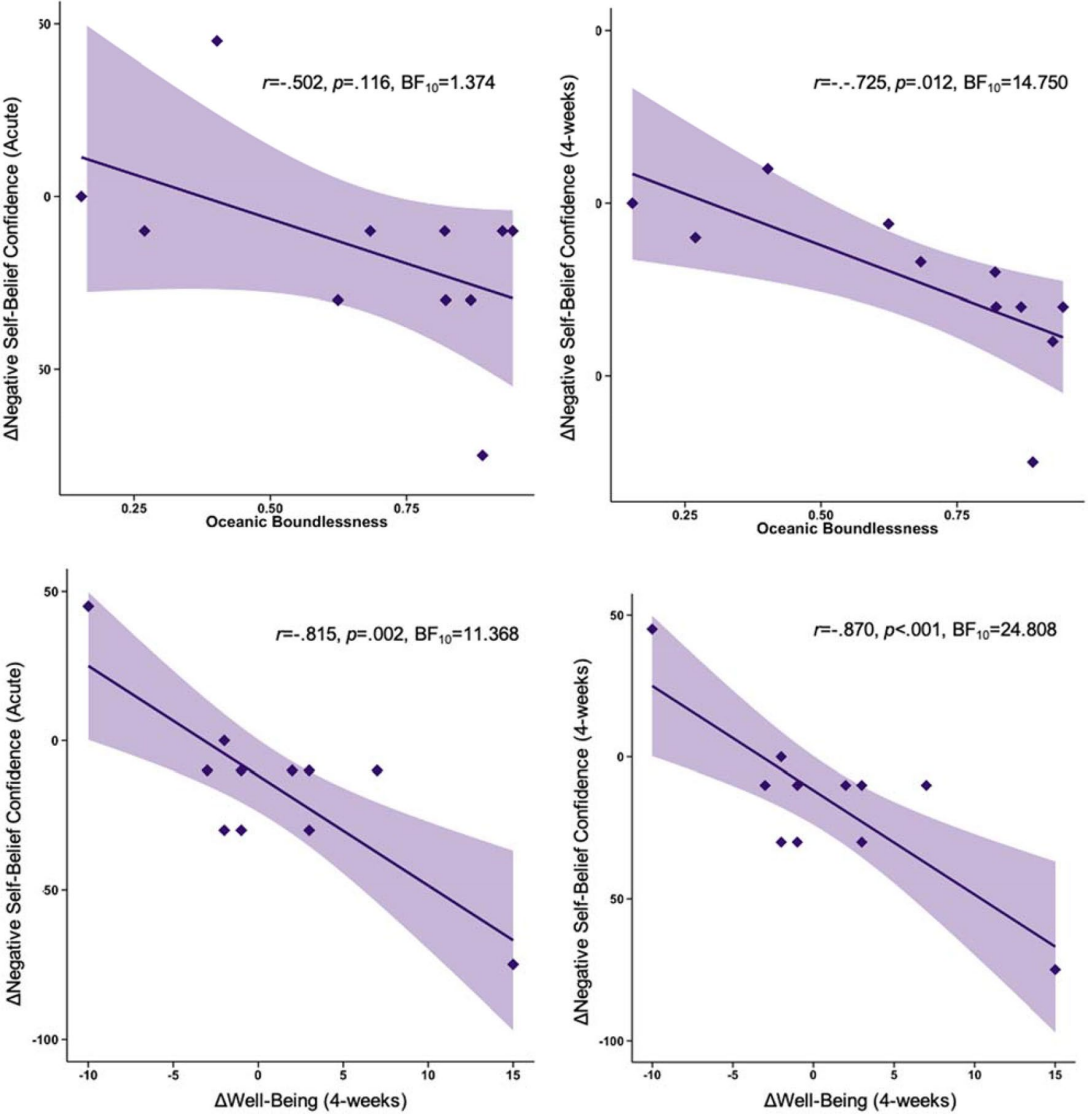
Association between decreases in negative self-belief confidence (acute and 4-week follow-up), acute oceanic boundlessness, and increases in well-being at 4-week follow-up. Shaded areas represent standard errors. ΔNegative Self-Belief Confidence (Acute) = Confidence at 4 h post-25 mg psilocybin– Confidence at pre-25 mg psilocybin. ΔNegative Self-Belief Confidence (4-weeks) = Confidence at 4-weeks post-25 mg psilocybin—confidence at pre-25 mg psilocybin.

Following the administration of 25 mg psilocybin, the effect size for the relationship between oceanic boundless and acute decreases in negative self-belief confidence was large (*r* = − 0.502; Fig. 2A), but not statistically significant (*p* = 0.116), with an anecdotal BF (BF_10_ = 1.374). Interestingly, this association grew over time, exhibiting a large (*r* = − 0.725; Fig. 2B) and significant (*p* = 0.012) effect 4-weeks after the session. The BF revealed substantial evidence towards the alternative hypothesis (BF_10_ = 4.750). In contrast, the relationship between oceanic boundlessness and decreases in acute negative self-belief confidence was small and non-significant both acutely (*r* = − 0.176, *p* = 0.604) and 4-weeks after 1 mg psilocybin (*r* = 0.048, *p* = 0.889). BFs were anecdotally in support of the null hypothesis (Acute BF_10_ = 0.655; 4-week BF_10_ = 0.601). See Fig. 2. For correlations between changes in negative self-belief confidence and additional ASC scales, see Supplementary Material.

### Changes in negative-self belief confidence and increases in well-being

Following 25 mg psilocybin, results showed a large (*r* = − 0.815) and significant (*p* = 0.002) association between decreases in acute negative self-belief confidence and increases in well-being 4-weeks later (Fig. 2C). Decreases in negative self-belief confidence measured at 4-week follow-up remained strongly associated with increases in well-being (*r* = − 0.870, *p* < 0.001; Fig. 2D). These findings were supported by strong BFs for the association between increases in well-being and decreases in negative self-belief confidence during the acute experience (BF_10_ = 11.368) and 4-weeks after the session (BF_10_ = 24.808).

For the 1 mg session, there was a small and non-significant association between decreases in acute negative self-belief confidence and decreases in well-being at 4-week follow-up (*r* = 0.178, *p* = 0.601). There was a moderate but non-significant association between increases in well-being and decreases in negative self-belief confidence at 4-week follow-up (*r* = 0.546, *p* = 0.082). The BFs were anecdotally in support of the null hypothesis for the association between changes in well-being and changes in negative self-belief confidence during the acute experience (BF_10_ = 0.656) and anecdotally in support of the alternative hypothesis at 4-weeks (BF_10_ = 1.645).

### Changes in negative-self belief confidence and neural entropy

**Figure 3:**
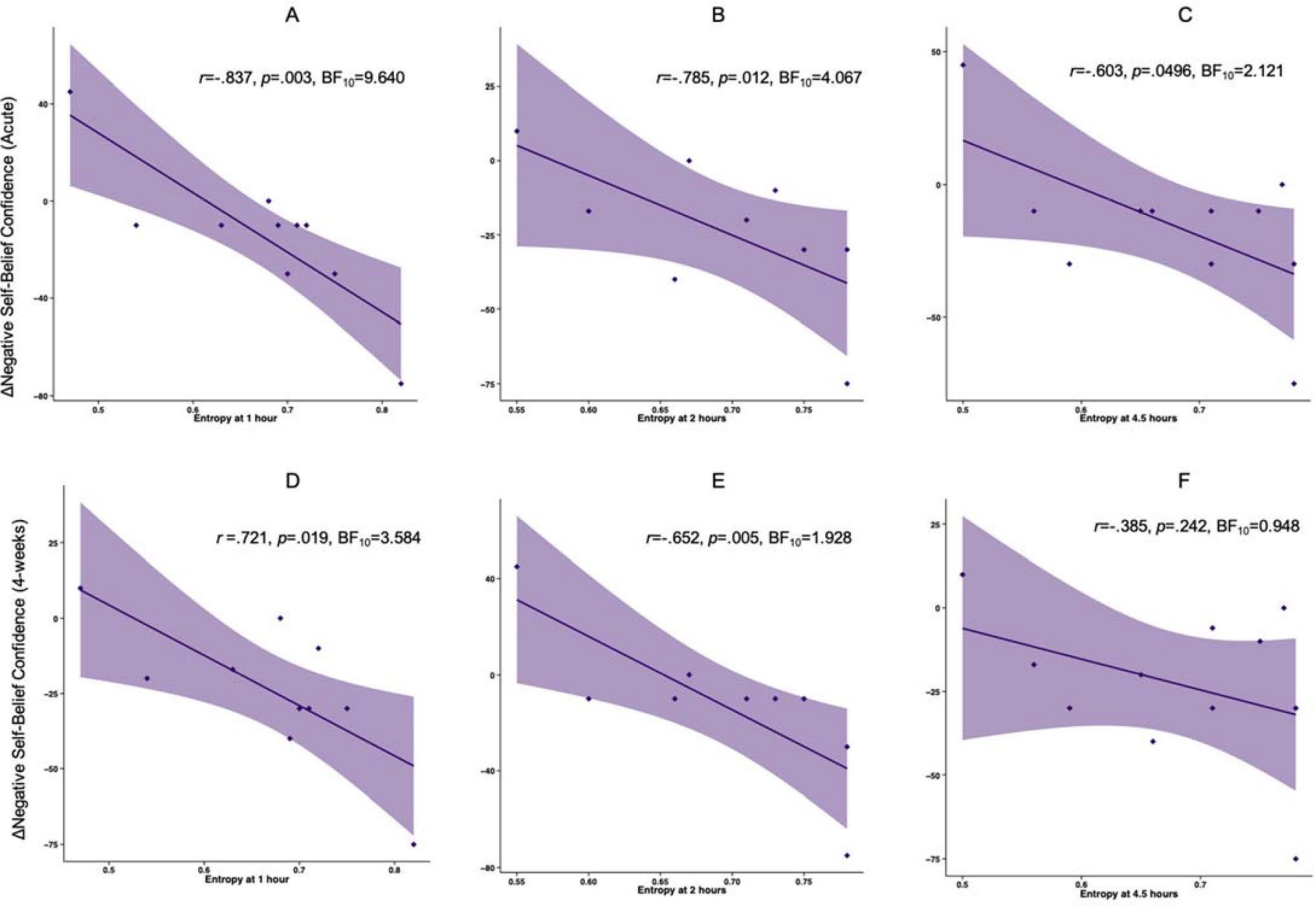
Association between decreases in negative self-belief confidence (acute and 4-week follow-up) and resting-state EEG entropy during the 25 mg psilocybin session. Shaded areas represent standard errors. ΔNegative Self-Belief Confidence (Acute) = Confidence ratings 4 h post-25 mg psilocybin—confidence at pre-25 mg psilocybin; ΔNegative Self-Belief Confidence (4-weeks) = Confidence at 4-weeks post-25 mg psilocybin—confidence at pre-25 mg psilocybin. (**A**,**D**) n = 10; (**B**,**E**) n = 9; (Figs. 3**C** and 3**F**), n = 11.

Large associations were found between decreases in acute negative self-belief confidence and neural entropy at 1 (*r* = − 0.837, *p* = 0.003; Fig. 3A), 2 (*r* = − 0.785, *p* = 0.012; Fig. 3B), and 4.5 (*r* = − 0.603, *p* = 0.0496; Fig. 3C) hours after 25 mg psilocybin. This was supported by substantial BFs for 1(BF_10_ = 9.640) and 2 (BF_10_ = 4.067) hours, and an anecdotal BF for 4.5 h (BF_10_ = 2.121) after 25 mg psilocybin. Similarly, there were medium to large associations between neural entropy at 1 (*r* = − 0.721, *p* = 0.019; Fig. 3D), 2 (*r* = − 0.652, *p* = 0.005; Fig. 3E), and 4.5 (*r* = − 0.385, *p* = 0.242; Fig. 3F) hours after 25 mg psilocybin and decreases in negative self-belief confidence 4-weeks later (with significance at 1 h after administration but not at 2 and 4.5 h). This was reflected in a substantial BF at 1 h (BF_10_ = 3.584), anecdotal/weak BF at 2 (BF_10_ = 1.928), and no evidence at 4.5 h (BF_10_ = 0.948).

The associations between decreases in acute negative self-belief confidence and neural entropy after 1 mg psilocybin and were small and not significant for 1 (*r* = − 0.078, *p* = 0.830), 2 (*r* = 0.250, *p* = 0.517), and 4.5 (*r* = − 0.024, *p* = 0.944) hours after the beginning of the session. Evidence was very anecdotal towards the null (1 h BF_10_ = 0.622, 2 h BF_10_ = 0.735, 4.5 h BF_10_ = 0.598). Similarly, the associations between acute neural entropy at 1 (*r* = − 0.371, *p* = 0.291), 2 (*r* = − 0.184, *p* = 0.636), and 4.5 (*r* = − 0.338, *p* = 0.310) hours after 1 mg psilocybin and decreases in negative self-belief confidence 4-weeks later were small to medium and not significant. Here, there was no evidence to either support or reject a relationship (1 h BF_10_ = 0.873; 2 h BF_10_ = 0.683; 4.5 h BF_10_ = 0.849).

## Discussion

The recently proposed REBUS model^20^ provides a unified account of the effects of psychedelics and the mechanism through which they can potentially facilitate therapeutic change. Neuroscientific evidence for the REBUS model is growing, but little research has directly examined the model’s psychological counterpart. This is an oversight, as the model hinges on its psychological validity to an equivalent extent (to its neurobiological validity). Here we conducted a preliminary examination of key components of the REBUS model, which suggest that (a) psychedelics allow for an acute *relaxation* and post-acute *revision* in confidence associated with strongly held beliefs, and (b) decreased confidence in maladaptive beliefs is associated with improvements in well-being and neurobiological markers of increased neural entropy.

### From belief relaxation to belief revision

Four weeks after the administration of 25 mg psilocybin, we found a moderate-substantial and significant decrease in confidence in negative self-beliefs. These results expand on past research showing significant decreases in pessimism (e.g.,^32^) and hopelessness^6^ following psilocybin therapy. Further, we found evidence against the 1 mg of psilocybin inducing a change in negative self-belief confidence (i.e., BFs ~ 0.3), particularly in the acute state. Additionally, we observed large and significant increases in positive self-belief confidence 4-weeks after the administration of 25 mg psilocybin, with a moderate BF. This result is consistent with suggestions that relaxed priors at the microscale level may also manifest in subjectively experienced strengthened beliefs^40^ and past research indicating that psychedelics increase positive beliefs about the self (e.g.,^31,34^). This study is the first to include pre-post measurement of positive self-beliefs or personally identified positive self-beliefs. While the acute decreases in confidence for both categories of self-belief were not statistically significant, the effect size for negative self-beliefs was moderate. The BFs were fairly equivocal, suggesting that, rather than the data supporting an absence of effect, the current data are not sufficient to accept or reject either hypothesis. It will therefore be important for future studies to explore whether inference can be improved by increasing the sample size.

We observed weak evidence toward acute decreases in negative other-belief confidence, as well as evidence against changes in negative other beliefs 4-weeks after 25 mg psilocybin. We also did not find changes in confidence associated with positive other-beliefs and evidence against a change 4-weeks post 1 mg psilocybin. It is interesting to note that this was the only belief category with no apparent decrease in belief confidence in the acute state. There was little room for increases in belief confidence due to ceiling effects, and the BF indicates that the data are only anecdotally in support of the null hypothesis (i.e., no change). Nevertheless, this is interesting because it suggests that belief relaxation is not global and it is consistent with the tendency for psychedelics to induce a sense of social connectedness^41^. Conversely, these results may be due to these beliefs not having been salient enough for significant changes in confidence to occur, as we did not direct participant's attention toward the individual they had identified a belief about during the psilocybin experience.

Although there were generally decreases in acute certainty following 25 mg psilocybin (except for positive-other beliefs), changes in confidence 4-weeks after 25 mg psilocybin differ between belief categories. While these post-acute changes were generally in an adaptive direction (i.e., decreases in negative belief confidence and increase in positive belief confidence), this does not necessarily demonstrate that psychedelics inherently generate positive belief changes. Nevertheless, sensitisation of the brain to new evidence through the relaxation of high-level priors may sometimes be beneficial in itself (i.e., independent of the valence of the new evidence), especially given that positive (or desirable) evidence is typically overweighted and negative (or undesirable) evidence is typically underweighted in healthy individuals^42^. It is also likely that the administration of psilocybin in a comfortable and emotionally supportive environment contributed to the positive nature of the confidence change. While specific manipulations of the context (e.g., administering a psychedelic in an adverse or unsupportive environment) may be ethically problematic, measurement of individual's perception of the context (e.g., their relationship with their guides/therapists^43^), grading the extent of environmental support (e.g., from low to very high), and naturalistic research, may aid in understanding the effects of context on belief relaxation and revision^44^.

### Belief relaxation, belief revision, and the unitive experience

Following the 25 mg, but not the 1 mg psilocybin session, we found a strong relationship between participants’ acute unitive experience and decreases in negative self-belief confidence (acutely and 4-weeks later). The unitive experience is a key predictor of psychedelic therapy outcomes^37^ and has been framed as a phenomenological “proxy” for the entropic psychedelic state during which belief confidence is relaxed^20,39^. The current results provide further support for this interpretation and suggest that the unitive experiences may be associated with positive therapeutic outcomes *because* they are characterized by an acute relaxation of, and potential revision of, belief structures that maintain psychopathology.

Interestingly, relative to acute reductions in negative self-belief confidence, decreases in confidence were greater 4-weeks after 25 mg psilocybin and more strongly associated with the intensity of participants’ unitive experience. This suggests that a yet uncaptured post-acute mediator may be implicated and it will be important for future research to explore the factors that facilitate the successful transition from REBUS to REBAS, such as the process of re-evaluating previously held beliefs or successfully integrating the psychedelic experience.

### Belief relaxation, belief revision, and increases in well-being

We found a large and significant association between decreases in negative self-belief confidence (acute *and* at 4-weeks) and increases in well-being 4-weeks after the administration of 25 mg psilocybin. This is the first study to provide evidence for the association between psilocybin-modulated acute decreases in negative self-belief confidence and long-term improvements in well-being. The findings are in line with research indicating that decreased pessimism is associated reductions in depression severity^32^ and provide further support for the role of the relaxation (REBUS) *and* revision (REBAS) of beliefs as putative mechanisms underlying the positive therapeutic effects associated with psychedelics.

Although our sample was comprised of healthy individuals, these finding suggest that psilocybin therapy may be especially promising for the treatment of psychiatric disorders characterized by especially rigid and inflexible negative self-beliefs, such as depression^45^ and anorexia (see^46,47^). Moreover, integrating psychedelic therapy within therapies that challenge and increase distance from such beliefs (e.g., cognitive behavioural therapies) may help to enhance the efficacy of psychedelic therapy (see^48–53^). Relatedly, identifying and examining confidence associated with salient maladaptive beliefs within the context of psychedelic therapy may help to more effectively target such maladaptive beliefs.

### Belief relaxation, belief revision, and neural entropy

Previous research has suggested that the entropy-enhancing capability of psychedelics may be important for the decreases in subjective confidence in personally-held beliefs^20^. Bridging the neurobiological underpinnings of the REBUS model with its psychological implications, we found medium-large associations between neural entropy and reductions in negative self-belief confidence during 25 mg psilocybin and 4-weeks later. Neural entropy at 1, 2, and 4.5 h post-25 mg psilocybin was significantly associated with acute decreases in negative self-belief confidence. Additionally, neural entropy at 1 and 2 h post-25 mg psilocybin was significantly associated with reductions in negative self-belief confidence 4-weeks later. Nonetheless, due to the small sample size, we are more inclined to draw inferences from the BFs, rather than frequentist p-values, to (cautiously) qualify that these results provide very preliminary evidence in support of the REBUS model. These results are the first to find evidence for an association between the effects of psychedelics on neural entropy and both acute and sustained decreases in negative self-belief confidence.

## Limitations and future directions

The present findings should be interpreted in line with their limitations; First, and most pertinent of which being the relatively small sample of ‘healthy’ volunteers who are less likely to have pathologically overweighted confidence in maladaptive negative self-beliefs than clinical samples^45^. Small samples are prone to overestimating effect sizes and poorer reproducibility^54^. Furthermore, due to the small sample size and exploratory nature of the present study, the present analyses were not corrected for multiple comparisons. Accordingly, confirmation of these results and their extrapolation to clinical contexts and psychiatric disorders characterized by negative self-beliefs (e.g., anorexia) must await replication in studies with both larger sample sizes and clinical samples. Such research will be necessary to increase the specificity of the REBUS model and to identify the specific types of maladaptive beliefs that psychedelics can reliably relax and revise, as well as for whom such changes may be beneficial or harmful (e.g., it is possible that destabilization of positive and/or negative self-related beliefs may be iatrogenic or require increased psychotherapeutic treatment^55^). Of note, even within a healthy sample, we observed a decrease in negative self-belief confidence that was associated with improved well-being, which speaks to the prophylactic potential of psychedelics. Second, the present findings may be limited by difficulties surrounding blinding due to psilocybin’s acute psychoactive effects^56^. We attempted to control for possible expectancy effects by only including psychedelic naive individuals and a very low dose (1 mg) psilocybin session. Nonetheless, it will be worthwhile for future research to measure the strength of participant blinding (e.g., see^57,58^). Additionally, using other acutely psychoactive pharmacological agents as controls and designs with additional variable doses of psilocybin will add specificity to the relationship between dosing and changes in belief confidence (including belief relaxation [REBUS], strengthening, and revision [REBAS]^20,40^). Identification of a “sufficient” dose will be important for therapeutic implementation. While the present results are generally consistent with the REBUS model, they may also be accounted for by alternative neural models of psychedelics’ mechanism of action, such as the Altered Beliefs Under pSsychedelics [ALBUS] model^40^; also see^59^. Finally, there is likely to be a psychological process (e.g., *re-evaluation* or *reflection* of one's beliefs) that facilitates the transition from an experience of REBUS to post-acute REBAS^43^ that has not yet been elucidated and will have implications for therapeutic application. In line with this hypothesis, several studies^60–62^ have found that changes in well-being are predicted by active engagement in behaviours to integrate one’s psychedelic experience (i.e., making meaning and applying insights from the experience in one’s life^63^). To help facilitate this process, integration sessions are typically included in psychedelic research^63^ and their importance is often emphasized by participants^64^.

To help future research address some of these questions, we utilised these preliminary results to further develop the Relaxed BEliefs Questionnaire (REB-Q^65^), which provides a structured approach to measuring the psychological assumptions of REBUS. The measure instruct participants to identify their core beliefs and provides an index of confidence change in these self-identified beliefs. The measure can be flexibly adapted for the specific study population, aims, and design. Working versions of the measure and manual are available online (https://doi.org/10.31234/osf.io/r597a), and we encourage feedback from those wanting to use it in their own studies.

## Conclusion

In summary, REBUS is a recently proposed model that aims to account for the acute action of psychedelics and their potential to catalyze therapeutic change. In line with the REBUS model, we found that administration of a 25 mg dose of psilocybin was associated with specific decreases in negative self-belief confidence. Following 25 mg psilocybin, we found that decreases in negative self-belief confidence (acutely and 4-weeks after 25 mg psilocybin) were strongly associated with participants’ acute unitive experience and increases in well-being 4-weeks later. These results provide preliminary support for the possibility that the therapeutic action of psychedelics may be linked to their relaxation, and subsequent revision of, overweighted beliefs. Additional research, including larger samples and that facilitated by use of the REB-Q, will be necessary to replicate and further elaborate on these findings.

## Methods

### Overview

This study was conducted as part of a larger trial focused on investigating long-term psychological and brain changes following a single 25 mg dose of psilocybin in healthy psychedelic naïve volunteers. Psilocybin (COMP360) was provided by COMPASS Pathways. The study received a favourable opinion from the NRES London-Surrey Research Ethics Committee and was carried out in accordance with Good Clinical Practice Guidelines. The National Institute for Health Research/Wellcome Trust Imperial Clinical Research Facility (ICRF) provided site-specific approvals, and a Home Office Licence was obtained for the storage and handling of psilocybin.

### Participants

Participants were 11 healthy psychedelic-naïve individuals (4 females; age *M* = 42 years, *SD* = 10.12). For participant demographics, see Table 2. Inclusion criteria were: (a) physically and mentally healthy; (b) between 18 and 85 years old; (c) understanding of the English language; no experience with a psychedelic in the prior 12 months (11 participants had no lifetime experience with a psychedelic). Exclusion criteria included the presence of (a) a current or previously diagnosed psychiatric disorder; (b) current medically significant condition that renders them unsuitable for the study (e.g., diabetes, severe cardiovascular disease); (c) positive pregnancy test at screening or during the study; (d) excessive use of alcohol or other drugs (determined by study physician); and (e) no email access. All participants provided written informed consent.

**Table 2:** Participant demographics.

Demographic	Category	N(%)	*M (SD)*
Age			42 (10.12)
Sex	Female	4 (36)	
	Male	7 (64)	
Nationality	United Kingdom	9 (82)	
	Israel	2 (18)	
Ethnicity	White British	8 (64)	
	Mixed other	2 (18)	
	Black African	1 (9)	
Employment	Unemployed	1 (9)	
	Part-time employment	1 (9)	
	Full-time employment	9 (82)	
Education	None	1 (9)	
	College Diploma	5 (45)	
	Associate Degree/Technical Degree	2 (18)	
	Doctorate or professional degree	3 (27)	

### Procedures

This study used a single-blind, fixed order, within-subjects design. Participants underwent two dosing sessions four weeks apart. The first session involved a 1 mg dose of psilocybin and the second, a 25 mg dose of psilocybin. Previous research has shown that 1 mg can be regarded as sub-perceptual/subthreshold for subjective effects and is regarded as an inactive or negligibly active dose, while 25 mg is the dose of choice in many therapeutic studies^1^. Participants were not informed of the dosing order or the psilocybin dosage for either dosing session. Dosing sessions were conducted in line with study protocols suggested by Johnson et al.^66^, including preparation prior to, and integration session following, dosing sessions. Psilocybin administration occurred in a comfortable and dimly lit environment, and included a preselected music playlist, eyeshades, and two ‘guides’ (therapeutic support persons) to provide comfort and reassurance. Throughout dosing days, participants gave hourly ratings of drug intensity from 0 (not at all) to 10 (most intense drug effect imaginable). The end-of-dosing test battery started at least 4 h post-administration and only once participants gave a drug intensity rating of 4/10 or less.

Resting-state electroencephalogram (EEG) was measured with a 24-channel wireless EEG head cap (DSI-24 System, Wearable Sensing; 0.317 μV resolution, 300 Hz sampling rate) with 21 active channels. Pz and FPz acted as reference and ground during recording respectively. EEG data were recorded (via Bluetooth using DSI-Streamer-v.1.08.41) during the 1 mg and 25 mg psilocybin sessions at the following time points: (a) baseline (prior to the administration of psilocybin), (b) 1-h post-dosing, (c) 2-h post-dosing, and (d) approximately 4.5 h post-dosing and only once participants gave a drug intensity rating of 4/10 or less. During the EEG resting state recordings relevant for this manuscript, participants were instructed to focus on their intentions for their dosing session and to remain still with their eyes closed for four minutes.

### Measures

#### Belief confidence

Idiographic beliefs and confidence associated with these beliefs were measured using the RElaxed Beliefs Questionnaire (REB-Q) developed for the present study (see Supplement). One day before the 1 mg psilocybin session (1 mg psilocybin baseline), participants were verbally instructed to identify: (a) a negative belief about themselves (negative self-belief); (b) a positive belief about themselves (positive self-belief); (c) a negative belief about a person they disliked (negative other-belief); (d) a positive belief about a person they loved (positive other-belief). In situations where participants found it difficult to identify one of these beliefs, they were guided by one of their guides to do so through examples and self-reflection. Participants were also asked to rate the extent to which they were confident that each belief was true on a scale from 0 (“Not at all certain”) to 100 (“Absolutely certain”) at five time points: (a) 1 mg psilocybin baseline; (b) during the 1 mg psilocybin session/4.5 h after psilocybin administration (acute 1 mg psilocybin); (c) 4-weeks after 1 mg psilocybin/one day before 25 mg psilocybin (25 mg psilocybin baseline); (d) during the 25 mg psilocybin session/4.5 h after psilocybin administration (acute 25 mg psilocybin); and (e) 4-weeks after 25 mg psilocybin.

#### Acute experience

Acute experiences were measured using the Altered States of Consciousness questionnaire (ASC)^67^. The oceanic boundlessness scale of the ASC was used as a measure of acute unitive experience and is the ASC scale most closely related to the positive therapeutic outcomes associated with psychedelics^68^. The scale measures the experience of unity, spiritual experience, blissful state, insightfulness, and disembodiment. Items were rated on a digitally-presented visual analogue scale from 0 (“no more than usual”) to 100 (“much more than usual”). The ASC was completed following the 1 and 25 mg psilocybin sessions and within 1 h of participants rating their acute belief confidence.

#### Well-being

Well-being was measured using the Warwick-Edinburgh Mental Well-being Scale (WEMWBS)^69^, a 14-item self-report measure of subjective hedonic and eudaimonic well-being over the past two weeks. The scale consists of positively worded items rated on a 5-point scale from 1 (“None of the time”) to 5 (“All of the time”). The WEMWBS was completed at (a) 1 mg psilocybin baseline, (b) 4-weeks after 1 mg psilocybin/25 mg psilocybin baseline, and (c) 4-weeks after 25 mg psilocybin.

#### Entropy of neural signals

EEG data were used to calculate neural entropy during the 1 and 25 mg psilocybin dosing sessions. EEG data were missing from one participant at the 1 h post-administration time point and from two participants at 2 h post-psilocybin administration (both 1 and 25 mg psilocybin). Data were manually cleaned, low-pass filtered at 100 Hz, and subsampled at 200 Hz. The entropy of a signal corresponds to its level of unpredictability or diversity (i.e., how many different “patterns” are present in the signal). There are multiple methods to estimate the entropy of a signal, the most common of which is via a quantity known as Lempel–Ziv complexity (LZc). Here, we estimated entropy using the Context Tree Weighting (CTW) algorithm (based on the method described in^70^), which is known to outperform LZc in terms of bias, variance, and data-efficiency^71^.

### Data analysis

We employed a combination of frequentist and Bayesian Hypothesis Testing for all analyses. To examine changes in confidence related to their identified beliefs during (acute) and 4-weeks after 1 and 25 mg psilocybin (Question #1), we conducted general linear model (GLM) repeated measures ANOVAs. Follow-up pairwise comparisons examined the relationship between the time point (acute and 4-weeks after psilocybin administration) and respective baseline. Effect sizes were calculated using Cohen’s *d*_z_ for repeated measures. Pearson correlation coefficients were calculated to examine the association between changes in belief confidence and both (a) acute unitive experiences (Question #2b) and (b) changes in well-being (Question #2c). Changes in belief confidence and well-being were calculated by subtracting scores at each time point by their respective baseline (i.e., 1 mg/25 mg psilocybin baseline). Exploratory analyses also examined the strength of the association (Pearson correlation coefficients) between entropy and changes in negative self-belief confidence (Question #2a). The alpha level indicating significance was set at *p* < 0.05. All frequentist analyses were conducted using IBM SPSS Statistics (Version 26).

The addition of Bayes Factors (BF) is relevant to the current analysis for two main reasons: (1) BFs represent the strength of the evidence *for or against* the null hypothesis, and (2) the strength of a BF scale with the evidence and can therefore be used with small sample sizes^72^. Bayesian ANOVA and Bayesian correlations were performed with default JZS and Jeffries-Beta priors, respectively. The further a BF is from 1, the stronger the evidence for either the model of interest (BF_10_ > 1) or the competing model (BF_10_ < 1). Rstudio (https://rstudio.com/) was used for generating figures and Bayesian analysis, using the packages ggplot2^73^ and Bayes Factor^74^, respectively.

## Supplementary Information

Supplementary Information.

## Data Availability

The data that support the findings of this study are available from the corresponding author, RJZ, upon reasonable request.
