# Supplementary material for: From relaxed beliefs under psychedelics (REBUS) to revised beliefs after psychedelics (REBAS)
Source: Sci Rep. 2025 Jan 29;15:3651. doi: 10.1038/s41598-023-28111-3 (PMC11779827; doi:10.1038/s41598-023-28111-3)
Supplement: Supplementary file 1 — Supplementary Information. [file 41598_2023_28111_MOESM1_ESM.docx]

**Supplement**

**Relaxed Beliefs Questionnaire (REB-Q)**

**Baseline - Measure**

Everyone has beliefs about *themselves* and *other people.* Sometimes we hold these beliefs with absolute certainty and sometimes we are less certain about them. We are going to ask you to identify (a) one negative *and* one positive belief that you have about yourself, (b) one positive belief that you have about someone that you love, and (c) one negative belief that you have about someone that you strongly dislike. As well, for each belief, we will ask you to identify the extent to which you feel certain about the belief on a scale from 0 (Not certain at all) to 100 (Absolutely certain).

**Self-Belief Negative**

Please identify a *negative* belief that you have about **yourself** (e.g., I am unlovable, I am worthless, I am a coward, I am a bad person, I am a failure).

**Belief:** ____________________________________

How certain are you that this belief is true? _____________

*Rated on a visual analogue scale from* *0 (Not certain at all) - 100 (Absolutely certain)*

**Self-Belief Positive**

Please identify a *positive* belief that you have about **yourself** (e.g., I am loved by my family, I am talented, I am successful, I am a good person, I am an interesting person).

**Belief:** ____________________________________

How certain are you that this belief is true? _____________

*Rated on a visual analogue scale from* *0 (Not certain at all) - 100 (Absolutely certain)*

**Others Belief - Love**

Please identify an individual that you love ___________.

With regards to this individual, please identify a *positive* belief that you have about them (e.g., they are supportive, they are trustworthy, they are good, they are accepting, they are kind).

**Belief:** ____________________________________

How certain are you that this belief is true? _____________

*Rated on a visual analogue scale from* *0 (Not certain at all) - 100 (Absolutely certain)*

**Others Belief - Dislike**

Please identify an individual that you strongly dislike __________.

With regards to this individual, please identify a *negative* belief that you have about them (e.g., they are nasty, they are unforgiving, they are devious, they are hostile, they are bad).

**Belief:** ____________________________________

How certain are you that this belief is true? _____________

*Rated on a visual analogue scale from* *0 (Not certain at all) - 100 (Absolutely certain)*

**Acute and Post Measure**

Previously, we asked you to identify (a) one negative *and* one positive belief that you have about yourself, (b) one positive belief that you have about someone that you love, and (c) one negative belief that you have about someone you strongly dislike. Please identify how certain you are **right now** that each of these beliefs is true.

**Self-Belief Negative:**

**Currently**, how certain are you **right now** that the belief that [**insert Negative Self-Belief from baseline**] is true?

*Rated on a visual analogue scale from* *0 (Not certain at all) - 100 (Absolutely certain)*

**Self-Belief Positive**:

**Currently**, how certain are you **right now** that the belief that [**insert Positive Self-Belief from baseline**] is true?

*Rated on a visual analogue scale from* *0 (Not certain at all) - 100 (Absolutely certain)*

**Other-Belief-Love:**

**Currently**, how certain are you **right now** that the belief that [**insert Other-Belief-Love from baseline**] about **[insert individual that they loved from baseline]** is true?

*Rated on a visual analogue scale from* *0 (Not certain at all) - 100 (Absolutely certain)*

**Other-Belief-Dislike:**

**Currently**, how certain are you **right now** that the belief that [**insert Other-Belief-Dislike from baseline**] about **[insert individual that they strongly disliked from baseline]** is true?

*Rated on a visual analogue scale from* *0 (Not certain at all) - 100 (Absolutely certain)*

**Additional questions at post measure**

*If you were to identify these beliefs* ***now****, do you think you would identify the same beliefs?*

Belief #1: [**Insert Belief 1 from baseline**] *(yes/no)*

*If no, what would your new negative belief about yourself be? __________*

How certain are you that this belief is true? _____________

*Rated on a visual analogue scale from* *0 (Not certain at all) - 100 (Absolutely certain)*

Belief #2: [**Insert Belief 2 from baseline**] *(yes/no)*

*If no, what would your new positive belief about yourself be?__________*

How certain are you that this belief is true? _____________

*Rated on a visual analogue scale from* *0 (Not certain at all) - 100 (Absolutely certain)*

Belief #3: [**Insert Belief 3 from baseline**] *(yes/no)*

*If no, what would your new belief about the person you love be?__________*

How certain are you that this belief is true? _____________

*Rated on a visual analogue scale from* *0 (Not certain at all) - 100 (Absolutely certain)*

Belief #4: [**Insert Belief 4 from baseline**] *(yes/no)*

*If no, what would your new belief about the person you dislike be?__________*

How certain are you that this belief is true? _____________

*Rated on a visual analogue scale from* *0 (Not certain at all) - 100 (Absolutely certain)*

**Changes in Negative-Self Belief Confidence Following 25 mg Psilocybin and Acute Experiences (ASC)**

Following the administration of 25 mg psilocybin, the relationship between acute decreases in negative self-belief confidence and (a) anxious ego dissolution (r=.154, p=.651, BF_10_=0.640); (b) visionary restructuralization (r=.338, p=.309, BF_10_=0.850); (c) auditory alterations (r=-.063, p=.853, BF_10_=0.604); and (d) reduction of vigilance (r=-.428, p=.189, BF_10_=1.072) was not statistically significant. The relationship between decreases in negative self-belief confidence 4-weeks after 25 mg psilocybin and (a) anxious ego dissolution (r=.200, p=.555, BF_10_=0.673); (b) visionary restructuralization (r=.350, p=.292, BF_10_=0.872); (c) auditory alterations (r=-.072, p=.833, BF_10_=0.606); and (d) reduction of vigilance (r=-.502, p=.116, BF_10_=4.750) were also not statistically significant. However, there was a statistically significant relationship between decreases in negative self-belief confidence 4-weeks after 25 mg psilocybin and reduction of vigilance (r=-.669, p=.0243), with substantial evidence for the alternative hypothesis (BF_10_=3.163).

Following the administration of 1 mg psilocybin, the relationship between acute decreases in negative self-belief confidence and (a) anxious ego dissolution (r=-.205, p=.546, BF_10_=0.677); (b) visionary restructuralization (r=-.334, p=.316, BF_10_=0.842); (c) auditory alterations (r=-.262, p=.437, BF_10_=0.734); and (d) reduction of vigilance (r=-.532, p=.092, BF_10_=1.551) was not statistically significant. Similarly, the relationship between decreases in negative self-belief confidence 4-weeks after 25 mg psilocybin and (a) anxious ego dissolution (r=0.069, p=.841, BF_10_=0.605); (b) visionary restructuralization (r=.091, p=.790, BF_10_=0.612); and (c) auditory alterations (r=.056, p=.869, BF_10_=0.602) were also not statistically significant. There was a statistically significant relationship between decreases in negative self-belief confidence 4-weeks after 1 mg psilocybin and reduction of vigilance (r=-.231, p=.049). However, there was nonetheless anecdotal evidence for the null hypothesis (BF_10_=0.701).
